# Development of response surface method prediction model for traffic-related roadside noise levels based on traffic characteristics

**DOI:** 10.1007/s11356-023-28934-7

**Published:** 2023-08-02

**Authors:** Ahmed Elkafoury, Bahaa Elboshy, Ahmed Mahmoud Darwish

**Affiliations:** 1 Department of Public Works Engineering, Faculty of Engineering, Tanta University, Tanta, 3111 Egypt; 2 Department of Architectural Engineering, Faculty of Engineering, Tanta University, Tanta, 31511 Egypt; 3 Transportation Engineering Department, Faculty of Engineering, Alexandria University, Alexandria, Egypt

**Keywords:** Response surface method (RSM), Traffic flow, Roadside noise levels, Optimization

## Abstract

Recently, several urban areas are trying to mitigate the environmental impacts of traffic, where noise pollution is one of the main consequences. Thus, studying the determinants of traffic-related noise generation and developing a model that predicts the level of noise by controlling the influencing factors are crucial for transportation planning purposes. This research aims at utilizing the response surface method (RSM) to develop a robust statistical prediction model of traffic-related noise levels and optimize different traffic characteristics’ ranges to reduce the expected noise levels. The results indicate that the rate of *L*_eq_ increase is higher at traffic flow values less than the 1204 veh/h. The interaction effect of flow-speed and flow-heavy vehicle percentage pairs shows that *L*_eq_ has peak values around 45.8 km/h and 28.71%, respectively, with almost symmetric value distribution about those center points. The main effects study indicates a direct effect of traffic flow, speed, density, and traffic composition on roadside noise levels. The prediction model has good representativeness of observed noise levels by predicted noise levels as the model has a high coefficient of determination (*R*^2^ = 95.87% and *R*^2^ adj = 92.26%) with a significance level of 0.0036. Then, the research presents a methodology to perform an optimization of the roadside noise level by defining traffic characteristics that can keep the noise level below 65 dB(A) or minimize noise level. Decision-makers could use the proposed method to control the roadside noise level.

## Introduction

Noise pollution has recently increased due to the huge increase in population density, construction activities, the demand for urban mobility in light of economic improvement, and the continuous growth in private vehicle ownership (Darwish et al. 2023). Road traffic is considered the most diffused source, especially in urban contexts, and needs monitoring, assessment, mapping, and prevention (Ruiz-Padillo et al. 2016). Thus, developing a suitable solution for noise pollution requires a holistic interpretation of problem elements and their cause required to help decision-makers develop the appropriate mechanisms to deal with such issues. Moreover, managing the level of noise needs developing a model that predicts the level of noise by controlling the influencing factors (Fredianelli et al. 2022).

Several models have been developed to predict the noise level and investigate the impact of different elements affecting it. These elements include population density, materials of pavements, and percentages of open spaces and green regions (Bianco et al. 2020; De Coensel et al. 2016; Gulliver et al. 2015; Suthanaya 2015). In addition, transportation is one of the most critical factors that cause noise in the streets (Steele 2001), where many related parameters affect noise emission (Praticò et al. 2021; Sandberg and Ejsmont 2002), but besides the engine and tire condition (Ramos-Romero et al. 2022; Teti et al. 2020), they comprise the flow composition (Filho et al. 2004), traffic volume, traffic density, and speed (Nicol and Wilson 2004). On the other hand, road pavement and surface layer have an impact on traffic noise generation (Bianco et al. 2020; de León et al. 2020; Del Pizzo et al. 2020; Praticò 2014; Praticò and Anfosso-Lédée 2012; Teti et al. 2020).

Different methods have been applied in modeling and predicting roadside noise. Table 1 shows a summary of the previous research studies. It clarifies the location, year, sample size, model type, and model accuracy.

**Table 1:** Review of previous research studies

SN	Reference	Location	Year	Methods	Independent variables	Dependent variables	Model accuracy, %
1	Kumar (2021)	India	2021	Response surface method (RSM) and artificial neural network (ANN)	Traffic volume, heavy vehicles percentage, and average speed	10-percentile exceeded sound level (*L*_10_) and equivalent continuous sound level (*L*_eq_).	*R*^2^ adjusted value 82.65~78.80%
2	Suthanaya (2015)	Denpaser City, Indonesia	2015	Multiple linear regression	Traffic volumes, speed, and road geometric	*L*_Aeq_, *L*_10_, *L*_50_, and *L*_90_.	*R*^2^ adjusted value 40.8~77.9% Average error value −2.33~+0.39%
3	(Lu et al. (2019)	Dalian City, China	2019	Structural equation modeling	Traffic volumes, number of lanes, and road segment length	*L*_*Aeq*_, *L*_10_, *L*_50_, and *L*_90_.	Root-mean-square error (RMSE) 0.031–0.084. Goodness-of-fit index (GFI) 0.993–0.999

Kumar (2021) applied the response surface method (RSM) and artificial neural network (ANN) to predict and improve traffic noise characteristics. Four models have been developed to determine the impact of traffic volume, heavy vehicles percentage, and average speed on the 10-percentile exceeded sound level (*L*_10_) and equivalent continuous sound level (*L*_eq_). The models used linear, square, interaction, and complete quadratic input variables. The entire quadratic model (4) showed the best performance in accuracy and prediction.

Suthanaya (2015) calibrated a model for road traffic noise in Denpasar City, Indonesia. The measurements were conducted along a collector road, as a case study, including classified traffic volumes, speed, and road geometric. The model was developed by the multiple linear regression technique. The models showed acceptable results with an average error varying between −2.33 and +0.39% between the predicted value and measured value for *L*_eq_, *L*_10_, *L*_50_, and *L*_90_.

Lu et al. (2019) explored the impact of road characteristics on traffic-related noise in Dalian City, China, using the structural equation modeling method. The models indicated high goodness of fit index values exceeding 0.99. The research showed that there is a high correlation between the lane number and traffic noise. Traffic noise increases with the increase in the number of lanes.

There are many ways to assess environmental noise levels. One of them is Common Noise Assessment Methods in Europe (CNOSSOS-EU) (Kephalopoulos et al. 2012). CNOSSOS-EU is developed for noise mapping, which is a set of guidelines and models developed by the European Union to assess environmental noise levels in Europe. It uses a standardized method for measuring and assessing noise levels and takes into account various factors such as noise sources, propagation, and the sensitivity of the human ear to different frequencies. CNOSSOS-EU provides a comprehensive and structured approach to noise assessment and can be used for various purposes such as developing noise maps and evaluating noise reduction measures. CNOSSOS-EU is primarily used for regulatory purposes in Europe.

On the other hand, response surface methodology (RSM) is a statistical technique used to optimize and model the relationship between a response variable (in this case, noise level) and several input variables (such as distance from the noise source, time of day). RSM uses mathematical models to predict noise levels based on various input parameters and can be used to optimize noise reduction strategies.

This research aims at utilizing the response surface method (RSM) to develop a robust statistical prediction model of traffic-related noise levels and optimize different traffic characteristics’ ranges to reduce the expected noise levels. The research presents a methodology to perform an optimization of the roadside noise level by defining traffic characteristics that can keep the noise level below 65 dB(A) or minimize noise level.

There are already well-known methods for the same purpose; however, modeling roadside noise is needed in any area, since it depends on local characteristics. That is, there is still a need for further investigation, especially in developing countries, into studying, modeling, and optimizing roadside noise generation. For example, the traffic composition in developing countries differs from developed countries (e.g., the level of heterogeneity). Thus, the adopted method is applied on a multilane divided roadway in New Borg El Arab City (NBC) in Egypt. The method is applied to investigate the effect of different traffic characteristics and reach general optimization of traffic-related roadside noise levels by controlling the major traffic characteristics. This prediction algorithm is to understand and solve other environmental impact problems of traffic movements.

This research paper is organized as follows: “Material and methodology”, after this introduction, presents the details of the research methodology. “Results and discussion” discusses the case study and data collection. “Conclusion” comprises the details of the experimental study. Section 5 discusses the results. Finally, Section 6 states the conclusion of this research.

## Material and methodology

Due to the presence of many influences and variables that affect the noise level, a comprehensive and efficient methodology that deals with such numerous factors has to be adopted to develop an accurate, robust, yet sensitive model. The RSM is a powerful tool for modeling and optimizing the effect of tractable factors on a response in complex phenomena. It is a broadly accepted limited cost statistical-based design of experiment and optimization method for parameters with a limited number of experiments (Georgiou et al. 2014; Yadav et al. 2014; Zhang et al. 2016). RSM introduces an analysis of the system as well as the influence of the individual independent variables and their interaction on the output of the system. The influence of the factors and different interaction dimensions and process optimization is illustrated in 2D or 3D plots (Mohammed and Jaber 2021).

**Fig. 1:**
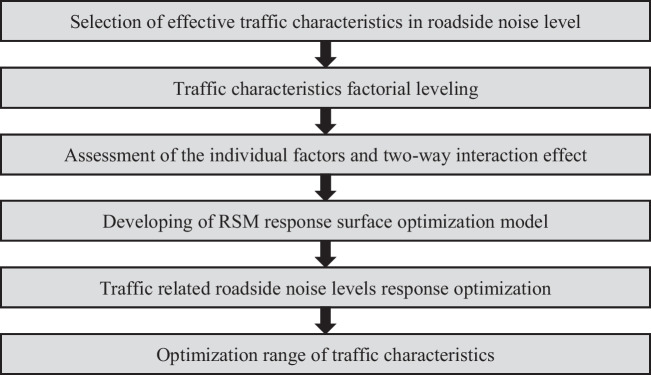
The optimization algorithm for an RSM of traffic-related roadside noise levels

In this research, RSM was performed using the MINITAB statistical analysis software. The targeted prediction model was developed based on the proposed optimization algorithm, illustrated in Fig. 1.

For acoustics related to architecture, roadway, and traffic applications, equivalent continuous sound level (*L*_eq_) is usually used to measure the ambient sound level as it minimizes the fluctuation in sound levels (Steele 2001; Suthanaya 2015). Thus, *L*_eq_ is used when there are quick fluctuations in noise that it is difficult to put an exact stable value on the noise level. Thus, it is useful to find the average sound level energy over some time as the equivalent continuous sound level that gives the same energy as the fluctuated sound level. Generally, *L*_eq_ is estimated by dividing sound exposure level (LE) by the time duration of the sound event, where LE is equivalent to the total sound energy.

To get a deeper assessment and understanding of the dependency of *L*_eq_ on different traffic characteristics response variables, the response surface analysis has been used. Generally, for measuring a quality characteristic, for example, the response surface, i.e. dependent variable (let it be *y*), is assumed to be in a relationship with other independent descriptive variables (let them be *x*1, *x*2, … , *xk*). This relation is expressed in Eq. 1.

1 $$y=f\left({x}_1,{x}_2,\dots .,{x}_k\right)+\xi$$

where *ξ* is a random variable and *k* is the number of independent variables. Suppose the tested parameters are in the vicinity of the optimal parameters, then the relationship between the quality characteristic and the independent factor becomes nonlinear. Thus, the second-order response surface model can be resolved in the form of a Montgomery model (Öktem et al. 2005; Saha and Biligiri 2017), as expressed in Eq. 2.

2 $$y={\beta}_0+\sum \nolimits_{i=1}^k{\beta}_i{x}_i+\sum \nolimits_{i=1}^k{\beta}_{ii}{x_i}^2+\sum \nolimits_{i\ne j}^k{\beta}_{ij}{x}_i{x}_j+\xi$$

where *βi* is the linear effect of *xi*, *βij* is the linear interaction effect between *xi* and *xj*, and *βii* is the secondary effect of *xi*.

The central composite design response surface modeling (CCD) is the most common statistical evaluation and design method associated with RSM (Öktem et al. 2005). CCD is suitable for RSM design where it is expected that the independent variables vary within a specific range (Öktem et al. 2005). In this research, and to characterize traffic-related noise levels, four main traffic characteristic factors (*F*, *HV*, *S*, and *K*) have been selected for consideration where:

- *F* indicates the traffic flow (number of vehicles passing a specific cross-section of the road link during a specific period) (veh/h),
- *HV* is the percentage of heavy vehicles (non-passenger cars) in the traffic composition,
- *S* is the average traffic speed (km/h), and
- *K* is the traffic density (number of vehicles that exists (moving or not moving) in a specific length of the road link) (veh/km).

In addition, the CCD is chosen as the RSM representative for the roadside traffic-related noise levels model (*L*_eq_) concerning the influence of the four independent traffic characteristics.

### Case study and data collection

New Borg El Arab City (NBC) is located 60 km to the southwest of Alexandria and 7 km away from the Mediterranean coast with 150,000 inhabitants. NBC is expected to reach 570,000 inhabitants by 2022. NBC was inaugurated in 1988 and is seen as the natural extension of Alexandria, Egypt (New and El 2015). It is regarded as the most important industrial zone in Egypt. It covers more than 2000 hectares and comprises about 1700 industrial facilities and institutions providing investors and owners with all the needed facilities and services.

Currently, NBC has major development plans underway; in addition, the current population of approximately 100,000 inhabitants is expected to grow up to 750,000 inhabitants by 2032. This context offered many opportunities for the sustainable development of the city and the surrounding industry in response to the environmental, social, and economic challenges that such expansion plans might arise (Antuña-Rozado et al. 2016).

**Fig. 2:**
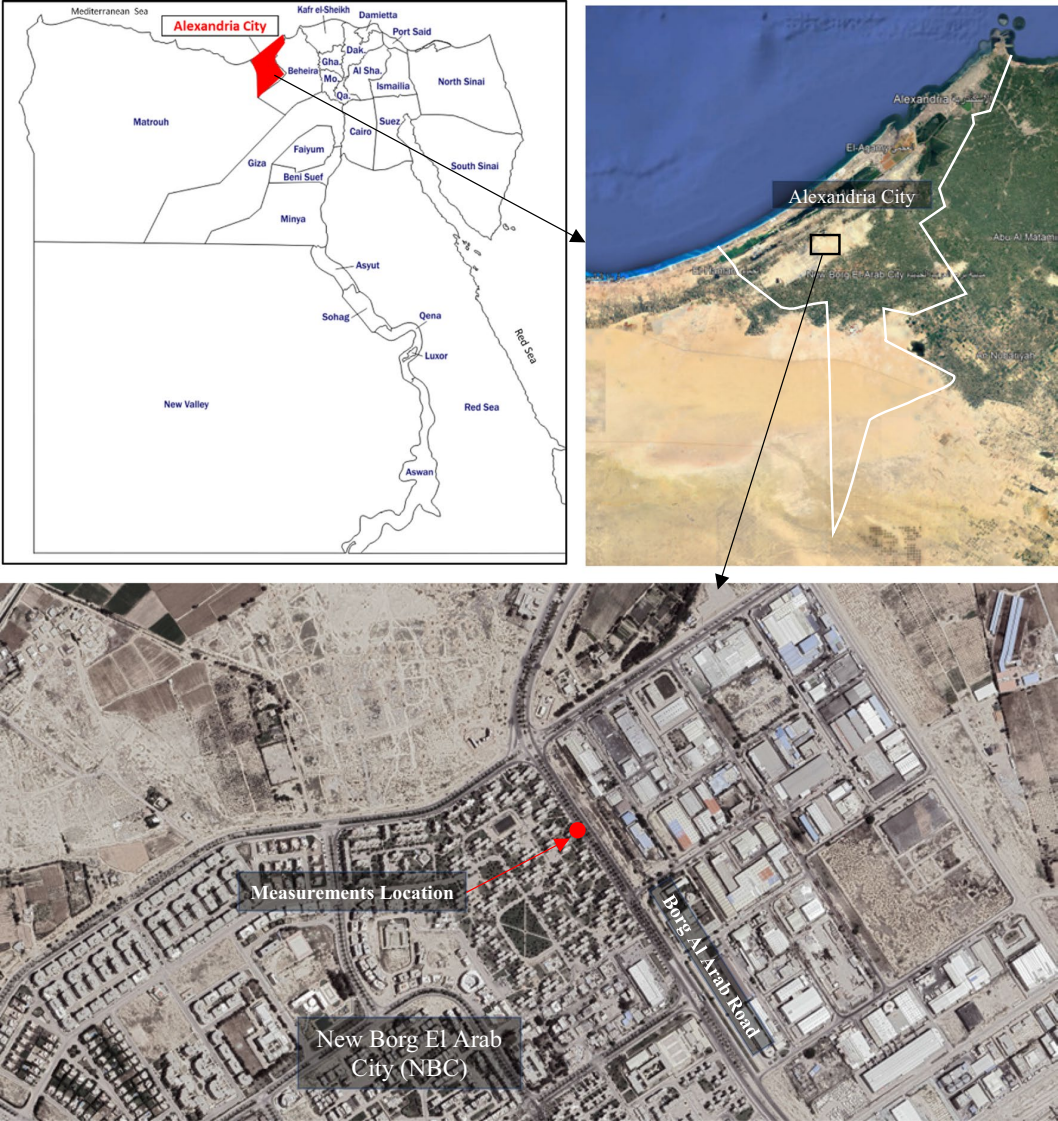
Selected link along a multilane divided roadway in NBC, Alexandria, Egypt

A multilane-divided roadway in NBC was selected as a case study, where the measurements of traffic characteristics and roadside noise levels were conducted. Field measurements were performed at a road cross-section that experiences dense traffic movement in the vicinity of the first industrial zone in the city. The site location is indicated in Fig. 2.

The measurements were held from 7:00 a.m. to 10:00 a.m. This time is selected to sample the noise levels during the morning peak traffic hours. The road cross-section comprises four asphalt paved lanes divided into two directions (7.5 m width of each direction) separated by a 4-m width concrete lane. Weather conditions were 22.7 °C air temperature, 5.76 km/h wind speed, and dry air as provided by the weather station in Egypt-Japan University of Science and Technology in NBC.

**Fig. 3:**
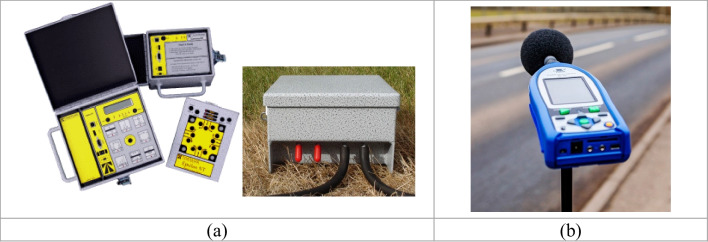
TimeMark Delta traffic analyzer (**a**) and Rion ambient sound analyzer (**b**)

The field instruments used in data collection are the traffic analyzer and ambient sound analyzer, as shown in Fig. 3.

For traffic data collection, TimeMark Delta — with rubber tube — traffic analyzer is utilized (Voigt et al. 2008). The analyzer counts the vehicle and classifies it based on the hits that occurred by the crossing vehicles on the rubber tube. The traffic analyzer comes with data processing software (VIAS2) that provides traffic characteristics for both directions, such as the number of vehicles, vehicle speed, and vehicle classification utilizing the Federal Highway Administration FHWA-F2A and the Basic-3A classification. In this study, a time interval of 1 min is set in the TimeMark instrument as the aggregation interval of the collected data. The analyzer was developed to classify passing vehicles, light vehicles (LVs), and heavy vehicles (HVs), including buses, single trucks, trucks, and trails. The output of the measurements was the traffic data for 4,221 vehicles.

Roadside noise levels, however, were measured using Rion sound level meters (Albitar and Bigazzi 2023), shown in Fig. 3. This type of device provides simultaneous measurements of sound pressure levels (Leq dB(A)) and allows the viewing of the stored data on a data processing software that comes with the device. The devices are set to measure the ambient sound levels every 1 min to match the same interval of traffic data collection. Before measurements, both the traffic analyzer and the sound level meters were calibrated according to their manuals.

### Experimental design

To perform the response surface analysis, the four traffic characteristics have been represented into three coded levels for each. The high level (+1), low level (−1), and center point (0) represent the maximum measured value, minimum measure value, and average value of each characteristic, respectively (see Table 2). Furthermore, 31 experimental runs were conducted to form the CCD matrix. Table 3 lists the values of responses at each of the 31 combinations of factorial levels generated by the principles of RSM.

**Table 2:** Coded levels for each traffic characteristic employed in RSM design

Level	Coded Level	*F* (veh/h)	*HV* (%)	*S* (km/h)	*K* (veh/km)
Low	−1	450	7.14	40	11
Center point	0	1204	28.71	45.8	27
High	+1	1740	86.67	51.2	39

**Table 3:** Experimental design and CCD matrix for *L*_eq_ RSM

Experiment No.	*F* (veh/h)	*HV* (%)	*S* (km/h)	*K* (veh/km)	*L*_eq_ dB(A)
1	0	0	0	1	59.8
2	1	−1	−1	−1	123.9
3	0	0	1	0	74.1
4	0	1	0	0	80.6
5	0	0	0	0	79.0
6	1	0	0	0	97.0
7	1	−1	1	1	72.3
8	0	0	0	0	79.0
9	−1	−1	1	1	28.9
10	0	0	0	0	79.0
11	−1	−1	−1	1	39.2
12	1	1	−1	−1	126.1
13	0	0	0	0	79.0
14	1	1	1	1	74.4
15	0	0	0	0	79.0
16	−1	1	−1	1	41.3
17	0	0	0	0	79.0
18	−1	−1	1	−1	70.4
19	0	0	0	−1	101.2
20	1	1	−1	1	84.6
21	1	−1	−1	1	82.5
22	−1	0	0	0	53.7
23	−1	1	−1	−1	82.7
24	0	0	−1	0	122.8
25	−1	−1	−1	−1	80.6
26	−1	1	1	−1	72.5
27	0	−1	0	0	78.4
28	0	0	0	0	79.0
29	1	−1	1	−1	113.7
30	−1	1	1	1	31.1
31	1	1	1	−1	115.9

#### Response variable assessment

**Fig. 4:**
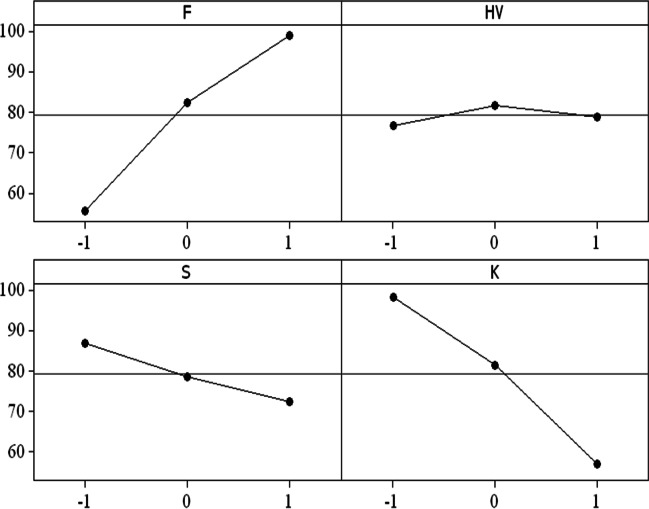
Main effect of traffic characteristics on Leq

Initially, the ANOVA analysis was utilized to assess the significance of each variable and the interaction of the variables in the linear and quadratic forms. A confidence interval of 95% is considered. Table 4 illustrates the analysis results of the significance of different traffic characteristics on traffic-related roadside noise levels. This indicates that all variables show significance to the noise levels in linear and quadratic forms, except for the %HV in all its forms. The interaction effects of any term involving %HV, as shown in Fig. 4, are found to have no significant impact on *L*_eq_.

**Table 4:** Significance of different traffic characteristics on traffic-related roadside noise levels

Term	Coef.	SE Coef.	*T*	*P*	Significance
Constant	3126.80	866.523	3.608	0.001	Significant
*F* (veh/h)	10.66	3.063	3.479	0.001	Significant
% HV	−3.29	2.551	−1.288	0.203	Insignificant
*S* (km/h)	−127.75	36.946	−3.458	0.001	Significant
*K* (veh/km)	−249.45	70.522	−3.537	0.001	Significant
*F* * *F*	0.00	0.001	3.391	0.001	Significant
% HV * % HV	0.00	0.002	1.092	0.279	Insignificant
*S* * *S*	1.34	0.395	3.384	0.001	Significant
*K* * *K*	5.07	1.466	3.460	0.001	Significant
*F* * % HV	−0.00	0.002	−1.222	0.227	Insignificant
*F* * *S*	−0.11	0.033	−3.417	0.001	Significant
*F* * *K*	−0.22	0.064	−3.426	0.001	Significant
% HV * S	0.07	0.055	1.230	0.224	Insignificant
% HV * K	0.11	0.082	1.297	0.200	Insignificant

#### The impact of traffic characteristics

The main effect study reveals a direction dependency of *F*, *HV*, *S*, and *K* on traffic-related roadside noise levels. The main effect plot in Fig. 4 elucidates the effect of each parameter on *L*_eq_. Furthermore, the percentage of heavy vehicles in traffic (HV) increases; *L*_eq_ initially slightly increases up to the center point level (28.71%), and then, *L*_eq_ slightly decreases back up to the high level of HV (86.67%). This brings up the fact that HV does not significantly affect traffic-related noise levels. Both traffic flow and density were found to significantly influence changes in noise levels.

In addition, *L*_eq_ values are found to monotonically increase with the increment increase of *F*. This result is consistent with the results of Nicol and Wilson (2004) and Saad and Alhiary (2005). However, the increase in traffic density is associated with a reduction in noise levels. This can be explained by the idea that traffic density represents traffic existence on a segment of the road link rather than traffic dynamics. In other words, as traffic density increases, vehicle movement decreases since the connections became more congested. As a result, traffic-related noise causes (tire–asphalt surface friction, air resistance, and motor sounds) are impacted. Regarding traffic speed, the upgrade of *S* values causes a significant reduction in noise levels in comparison to traffic density. These results are consistent with the coefficient results from the ANOVA analysis results (Table 4) where *F* has a positive coefficient of 10.66, while *K* and S have a negative coefficient of −249.45 and −127.75, respectively, which explains the larger impact of *K* on *L*_eq_ compared to *S*.

#### Two-way interaction effect

**Fig. 5:**
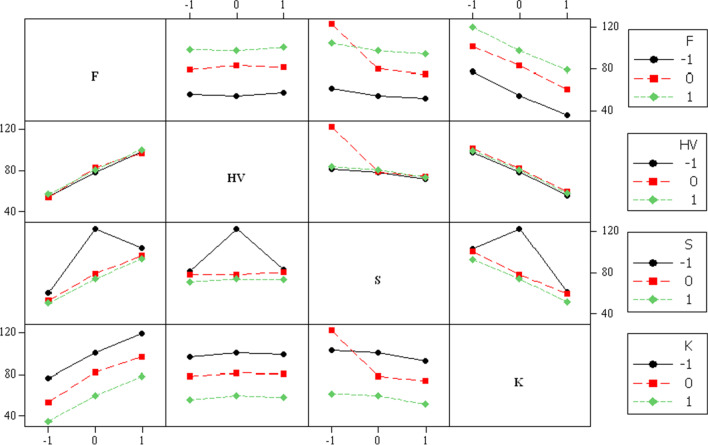
Interaction effect of pairs of traffic characteristics on Leq

ANOVA analysis also shows a considerable effect of the quadratic impact of the interaction between *S*, *K*, and *F*. This effect can be explained by the two-way interaction effects, shown in Fig. 5. Overall, traffic-related noise levels increased with the increase of *F* values. The interaction effect between *F* and *K* is found to be more significant in reducing *L*_eq_ compared to the interaction between *F*-*S* pairs. It is also shown that *L*_eq_ has an insignificant effect on *F*-*HV* interaction. The association of *HV* with other traffic characteristics is illustrated in the second row of Fig. 5. For all *HV* levels, the interaction between *HV* and any characteristic is indistinctive except with the *HV*-*S* interaction, which shows higher values of *L*_eq_ at the center point level of *HV* at lower levels of traffic speed. The significant trends of *HV*-*F* and *HV*-*K* interactions have similar values and different directions. In other words, *L*_eq_ values are found to increase with the increase of traffic flow values and decrease with the decrease of traffic density values for all levels of *HV* in *HV*-*F* and *HV*-K two-way interactions, respectively.

Regarding the interaction effect between traffic speed and any of the traffic characteristics, the significant effects at high and center point levels of *S* are identical. Overall, at *S* = 40 km/h, the interaction between *S* and other traffic characteristics (*F*, *HV*, and *K*) increases when *L*_eq_ values are at the center point levels of *F*, *HV*, and *K*. Then, *L*_eq_ values decreased. Comparing *L*_eq_ values at low traffic speed level (*S* = 40 km/h) to center point speed level (*S* = 45.8 km/h) shows that *L*_eq_ values at the center point levels of *F*, *HV*, and *K* experience significant reduction for all two-way interactions between *S* and traffic characteristics. The fourth row of Fig. 5 shows a significant increase in *L*_eq_ values as traffic density levels decrease. *K*-*HV* two-way interaction has no significant effect on traffic-related noise levels. Though *K*-*F* interaction announces a significant increase in *L*_eq_ values, *K*-*S* interaction shows a minor effect in reducing *L*_eq_ values.

#### Multiple response optimizations

**Fig. 6:**
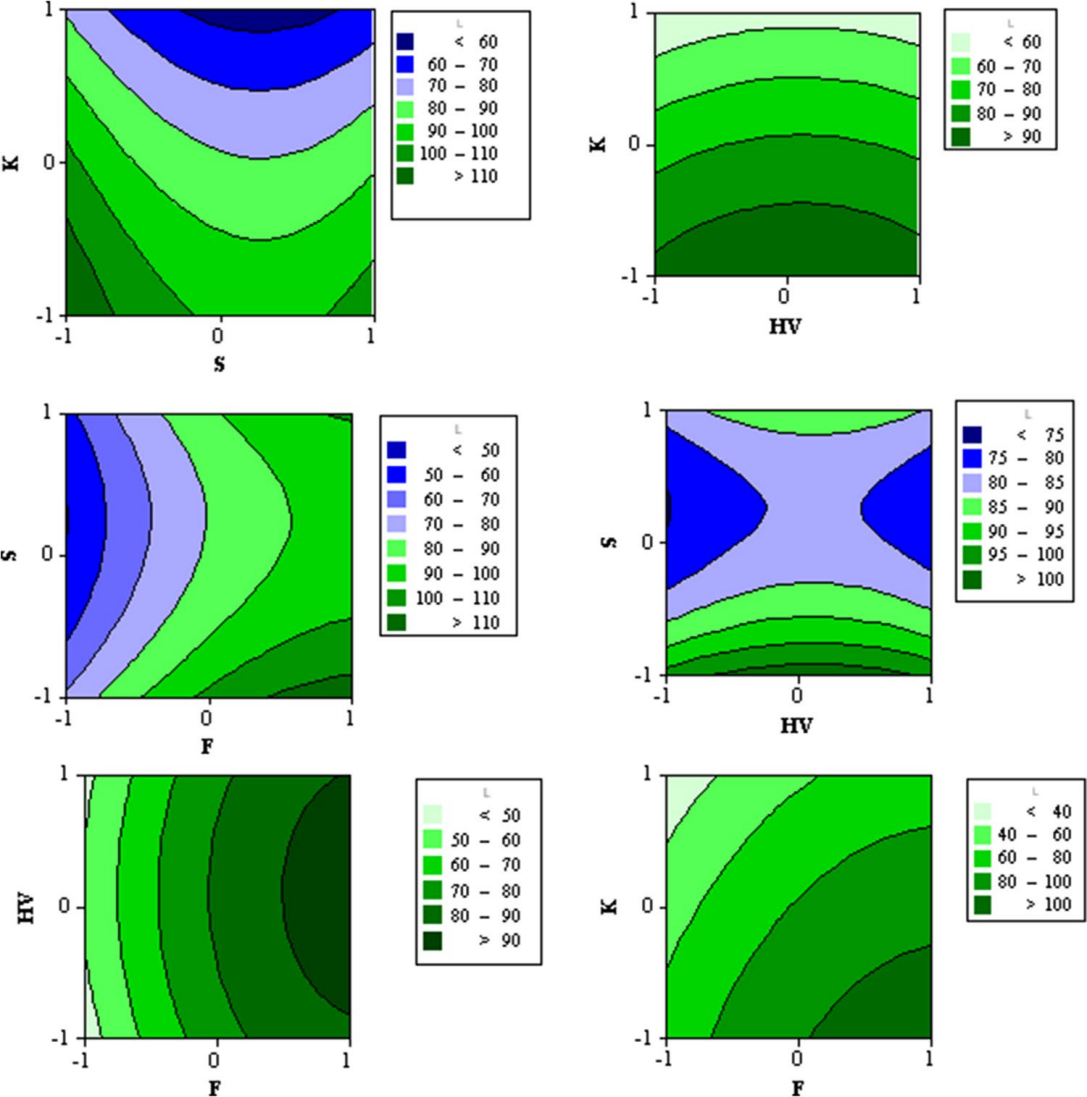
Contour maps of L_eq_ as a response to different values of pairs of traffic characteristics

When the two-way interaction between pairs of independent variables is statistically significant, contour plots give a complete view regarding this effect on *L*_eq_ (Khodaii et al. 2013). Multiple contour maps of the roadside traffic-related noise levels are presented in Fig. 6 to provide a better understanding of the changes in *L*_eq_ simultaneously with the changes in values of pairs of traffic characteristics and their mutual interaction. As shown in the figure, the increase of *K* from the low level (11 veh/km) to the center point level (27 veh/km) reduces *L*_eq_ for different levels of traffic speed. Changing *K* from 27 to 39 veh/km brings a steeper reduction in *L*_eq_ with various levels of traffic speed. Horizontally, with the increase in traffic speed, the noise levels slightly increase to the peak at the center point level of speed; then, the noise levels slightly decrease with the increase in speed values. Therefore, *K* is more significant than *S* in the value of traffic-related noise levels. The same behavior is noticed in the contour map of *HV*-*K* pairs. With the increase of *HV* values, the noise levels increment to the peak at the center point level of *HV* (28.71%); then, noise levels experience decrements with the increasing *HV* values. Also, it is found that for any HV value, the rate of reduction in noise levels with the increase of *K* levels is constant along with the whole range of *K*.

The contour maps of *F*-*S* and *F*-*HV* pairs illustrate that the increase of *L*_eq_ values correlates with the increase in traffic flow. It is noticed that the rate of *L*_eq_ increase is higher at traffic flow values less than the center level (1204 veh/h). For *F*-*S* and *F*-*HV* pairs, *L*_eq_ has its peak values around the center point of *S*, 45.8 km/h, and HV, 28.71%, with almost symmetric value distribution across those center points. Therefore, any traffic speed value or heavy vehicle percentages can distinguish the traffic flow effect on traffic-related noise levels. *F*-*K* contour map reveals that *L*_eq_ values increase diagonally by reducing *K* levels and increasing *F* levels. The diagonal rate of increase is almost linear. *HV*-*S* contour map shows that the highest *L*_eq_ values are noticed for the points with the lowest *S* levels and the center point *HV* levels. At the center point, *HV* level *L*_eq_ values decrease with an increase in traffic speed; then, at the highest speed values, *L*_eq_ slightly increases again. In addition, the lowest *L*_eq_ values are noticed at both the lowest and highest *HV* levels with center point *S* levels. It is also noted that *HV*-*S* points, where *L*_eq_ values range between 80 and 85 dB(A), occupy a large area in the contour map.

## Results and discussion

A quantifying surface response model was developed using the optimal predictor quadratic form for RSM optimization, based on Eq. 2, to optimize roadside traffic-related noise levels and obtain optimal responses. The model form is a second-degree polynomial function as shown in Eq. 3.

3 $$L\textrm{eq}=1016.3+0.07F+0.23 HV-41.56S-0.03K-0.001{F}^2-0.001{HV}^2+0.44{S}^2-0.03{K}^2$$

The prediction model has good representativeness of the observed noise levels since the model has a high coefficient of determination (*R*^2^ = 95.87% and *R*^2^ adj = 92.26%) with a model significance level of 0.0036 and a root-mean-square error (RMSE) of 0.76. This shows that the fitting model can express more than 92% of the response value with a high fitting level.

**Fig. 7:**
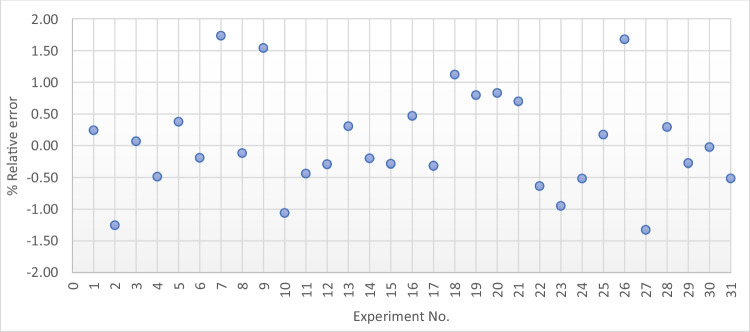
Scatter plot of the % relative errors between measured and predicted L_eq_ values for the RSM quadratic prediction model

To evaluate the accuracy of the models, a percentage of relative errors between the predicted *L*_eq_ and measured *L*_eq_ values was estimated for each of the experiments. If the model is appropriately selected, the percentage of the relative errors is minor. The RSM quadratic model results, shown in Fig. 7, illustrate that the errors are relatively low and almost equally distributed around the zero error. The maximum percentage of relative error recorded is 1.73%, with an overall average relative error of 0.05%. This all indicates that the models are acceptable.

The optimization process in this research is centered on the composite desirability of 1. The composite desirability is an objective function that ranges from zero, the outside limit to 1, the goal. The proposed second-order polynomial model is usually used to interpolate within the levels of the four studied factors. Thus, the RSM optimization tends to maximize the desirability function, starting from a random starting point of the independent variables, and repeating the starting point till finding the local maximum of the composite desirability (Chattoraj et al. 2014).

**Fig. 8:**
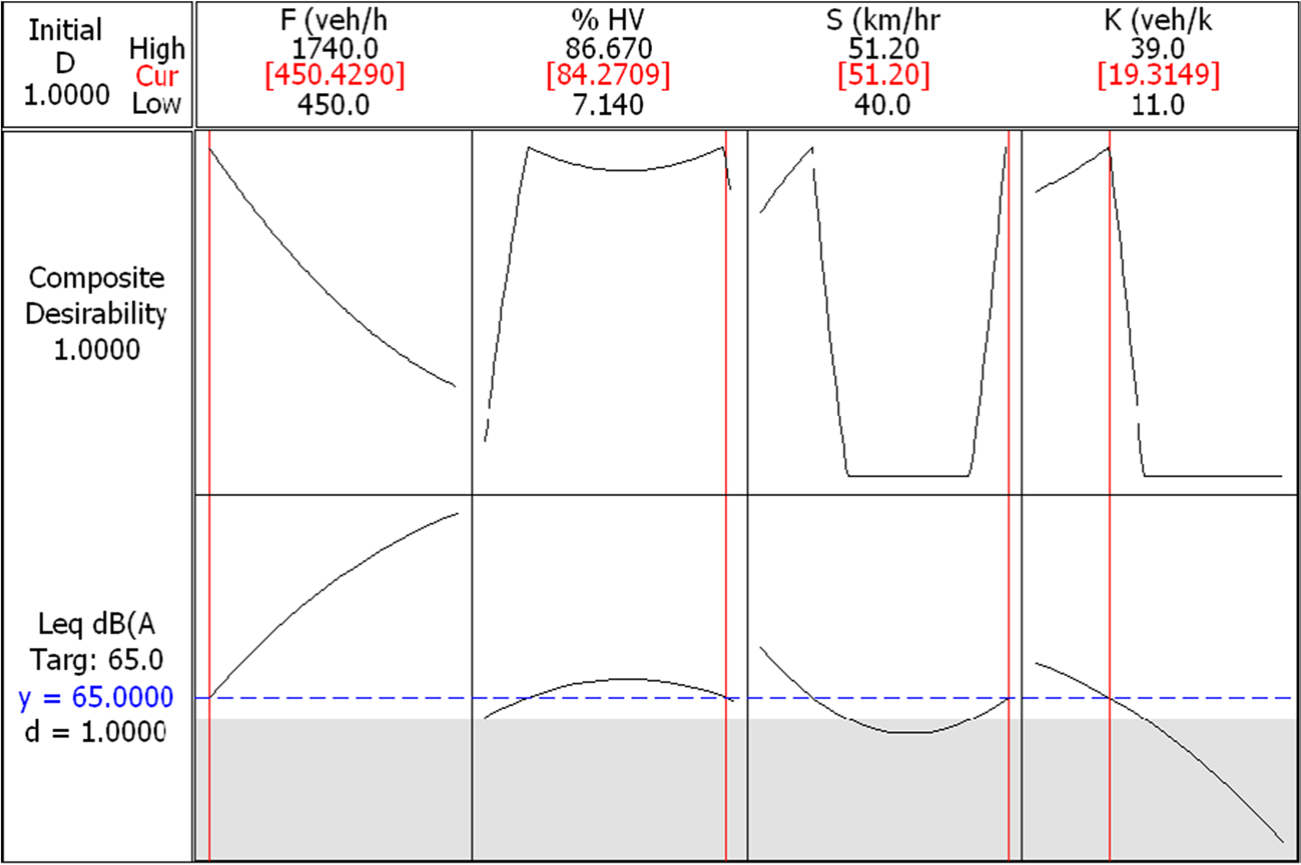
*L*_eq_ optimization results for a target value of 65 dB(A)

In this research, two optimization methods for traffic-related noise levels are considered. First, a target problem is predefined, where the target is the allowable residential noise level which is 65 dB(A), as illustrated in Fig. 8. Here, it is essential to mention that the center point values act as the pivot values of the composite desirability optimization process used in RSM optimization. It is found that 65 dB(A) traffic-related noise levels (A) are achieved at a traffic flow of 450 veh/h coupled with a percentage of the heavy vehicle of about 84%. Since the traffic density increments reduce noise levels, the optimization problem indicates that the target value allows a *K* value of about 19.3 veh/km. The target value is also hit at the highest level of speed (i.e., 51.2 km/h).

**Fig. 9:**
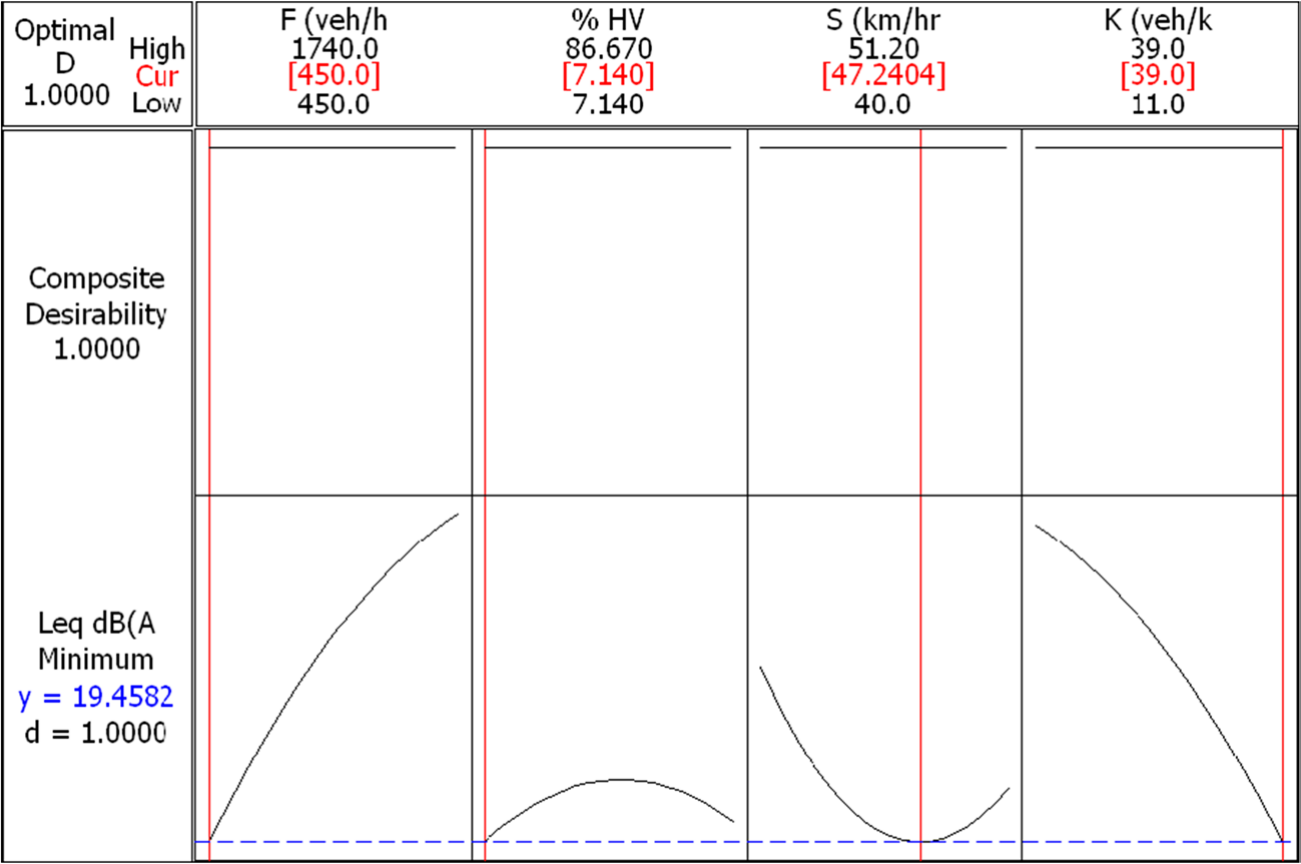
*L*_eq_ optimization results for a target of minimizing noise level

Second, the minimization noise level target is set, where the independent variables are negotiated to minimize the noise levels, as shown in Fig. 9. It is found that a traffic flow of 450 veh/h (highest level) coupled with a percentage of the heavy vehicle of about 7.14% (lowest level) produces the minimum response for all traffic speed and density values. Furthermore, a traffic speed of about 51 km/h at a traffic density of 39 veh/km produces the lowest response. Coinciding with the response trend in Figs. 5 and 6, it can be understood that the traffic speed in the range of 45–50 km/h shows a non-significant change in traffic-related noise levels.

Finally, a comparison is conducted with the results of other studies in the literature to justify the results as well as the methodology. The results indicate that the prediction model has good representativeness, where the model has a high coefficient of determination (*R*^2^ = 95.87% and *R*^2^ adj = 92.26%) with a model significance level of 0.0036. If compared with the developed models in the literature (refer to Table 1), the accuracy of the developed model can be considered superior, where the adjusted *R*^2^ in the literature models varied between 40.8 and 82.65%.

On the other hand, a validation is conducted through comparing the noise levels modeled by the developed RSM against noise levels modeled by CNOSSOS-EU method (Kephalopoulos et al. 2012). CNOSSOS includes methods for:

- Calculating noise levels
- Estimating the effects of noise on human health and the environment
- Developing noise management plans

The methods in CNOSSOS-EU are based on the best available scientific knowledge and are designed to be as flexible as possible to allow for different noise sources and settings. CNOSSOS-EU is an important tool for improving the management of noise pollution in Europe. It provides a common framework for noise assessment that can be used to compare noise levels across different countries and to develop effective noise management plans.

**Fig. 10:**
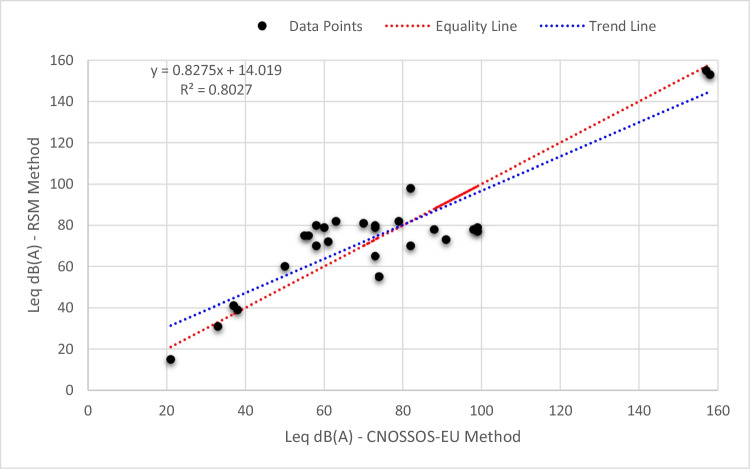
Comparison of noise levels modeled by the developed RSM against noise levels modeled by CNOSSOS-EU

According to Fig. 10, there is a good correlation between noise levels modeled by the developed RSM and noise levels modeled by CNOSSOS-EU with a correlation coefficient of 0.8027. Overall, the average percentage of error and its standard deviation are found to be 5.7% and 13.1%, respectively. These values are considered acceptable and indicate good match of the representativeness of the outputs.

## Conclusion

This research studies the effect of different traffic characteristics on the level of noise on roadsides using the response surface analysis. The main contribution of this research is the use of RSM methodology to form a prediction model for the level of noise on the roadsides. In this mode, it has been possible to reach the optimum limits for each of the traffic characteristics, included in the study.

The four characteristics of the traffic that affect roadside traffic-related noise levels are traffic flow, percentage of heavy vehicles (non-passenger cars) in the traffic composition, average traffic speed, and traffic density. The experiment was designed by classifying each of these characteristics into three levels to perform the response surface analysis. The ANOVA analysis is utilized to assess the significance of each variable and the interaction of the variables on the linear and quadratic forms. A confidence interval of 95% is considered. The main effects studied reveal a dependency direction of *F*, %*HV*, *S*, and *K* on traffic-related roadside noise levels. The prediction model has good representativeness of the observed noise levels by the predicted noise levels since the model has a high coefficient of determination (*R*^2^ = 95.87% and *R*^2^ adj = 92.26%) with a model significance level of 0.0036. This shows that the fitting model can express more than 92% of the response value with a high fitting level.

The study presented a methodology to perform an optimization of the roadside-related traffic noise level that can be implemented in the control of the noise level process in addition to preliminary studies in land use planning. To this end, two optimization targets have been predefined and analyzed to assess their achievability. The first target is capping the noise level to 65 dB(A), while the second target is minimizing noise levels. The results demonstrated that the first target is achieved in three situations: traffic flow of 450 veh/h coupled with a percentage of the heavy vehicle of about 84%, *K* value of about 19.3 veh/km, or speed of 51.2 km/h. The two cases achieve minimized noise levels at either traffic flow of 450 veh/h (highest level) coupled with a percentage of heavy vehicles of about 7.14% (lowest level), or traffic speed of about 51 km/h at a traffic density of 39 veh/km.

For future work, it is recommended to increase the number of measurements and conduct the surveys at different locations. On the other hand, model development can be enhanced with the use of artificial intelligence (AI) methods.

## Data Availability

The data that support the findings of this study are available from the corresponding author, upon reasonable request.
